# Exposures Associated with Non-Typhoidal *Salmonella* Infections Caused by Newport, Javiana, and Mississippi Serotypes in Tennessee, 2013–2015: A Case-Case Analysis

**DOI:** 10.3390/pathogens9020078

**Published:** 2020-01-24

**Authors:** Nabanita Mukherjee, Vikki G. Nolan, John R. Dunn, Pratik Banerjee

**Affiliations:** 1 Division of Epidemiology, Biostatistics, and Environmental Health, School of Public Health, University of Memphis, Memphis, TN 38152, USA; nmkhrje1@memphis.edu (N.M.); vgnolan@memphis.edu (V.G.N.); 2 Department of Infectious Diseases, Center of Excellence for Influenza Research and Surveillance (CEIRS), St. Jude Children’s Research Hospital, Memphis, TN 38105, USA; 3 Communicable and Environmental Diseases and Emergency Preparedness, Tennessee Department of Health, Nashville, TN 37243, USA; john.dunn@tn.gov

**Keywords:** salmonellosis, non-typhoidal *Salmonella* (NTS), *S*. Newport, *S*. Javiana, *S*. Mississippi, case-case analysis

## Abstract

Non-typhoidal *Salmonella* (NTS) infection (salmonellosis) is one of the most prevalent gastrointestinal diseases throughout the world. Human infections caused by *Salmonella* Newport, Javiana, and Mississippi serotypes have been observed to occur at higher rates on an annual basis in western Tennessee. The reason for the increased rate of NTS infection by these three serotypes in this region is not known. We conducted a case-case analysis to identify potential risk factors associated with the three *Salmonella* serotypes using FoodNet data, obtained from the Tennessee Department of Health, consisting of 1578 culture-confirmed salmonellosis cases in Tennessee from 2013 through 2015. Among all the exposure variables tested (254 in total), we found contact with pet treats or chews in the seven days prior to illness was the factor that was significantly associated with these serotypes compared to other serotypes (odds ratio _adjusted_ = 3.0 (95% confidence intervals 1.6, 5.5), *P* < 0.0005). This study highlights the need for further investigation of potential exposures (other than pet treats or chews), including several possible environmental sources of NTS infection in humans.

## 1. Introduction

Salmonellosis or non-typhoidal *Salmonella* (NTS) infections are common and significant public health concerns in the USA. The transmission of NTS in humans occurs through the ingestion of contaminated food and environmental exposures [1]. Common food sources of NTS infections include cheese made from unpasteurized milk, raw vegetables, undercooked beef, pork, eggs, and other poultry products [2,3,4,5]. Although rare, there are some reports of transmission of NTS infections via water [6]. In addition to food and water, several outbreaks suggest a strong association of NTS infections with direct or indirect contact with infected animals or their environment [7,8,9]. Many animals are asymptomatic carriers of *Salmonella* spp. and, thus, may serve as important reservoirs of NTS infections. Reptiles and amphibians are well-known carriers of several *Salmonella* serotypes, including, *S.* Newport and *S.* Javiana [7,10].

Recently, several studies established increased rates of NTS infections, particularly infections by *S*. Newport, *S*. Javiana, and *S.* Mississippi, in southern and southeastern United States each year [6,11,12,13,14]. In Tennessee, these infections have been observed to occur at higher rates in western counties during 2010–2014 [15]. Microbiological findings revealed an increased incidence of *S*. Newport, *S*. Javiana, and *S*. Mississippi in western Tennessee counties compared to other Tennessee counties [15]. Interestingly, the same trend was also reported in Louisiana, according to a report from the Louisiana Office of Public Health (LPH) [16]. Based on the similarity of these findings annually for these serotypes [15,16], we hypothesized that certain exposure signals in the geographical area of *S*. Newport, *S*. Javiana, and *S*. Mississippi clustering might be identified via case-case analysis. A common feature of these three serotypes is that historically they are associated with non-foodborne exposures, such as animals (pets and pests/rodent), birds (domestic or wild/migratory), amphibians, reptiles, and other aquatic organisms, or abiotic environmental components, such as soil, water, and dust [2,17,18,19]. Additionally, most of these infections are pan-susceptible which supports environmental exposure rather than exposure to food animal sources. This study analyzed existing data to assess associations of food, water, animal, and environmental exposures among salmonellosis cases in the state of Tennessee, USA, caused by *S.* Newport, *S*. Javiana, and *S*. Mississippi during 2013–2015.

## 2. Materials and Methods

### 2.1. Data Source

The data analyzed were collected from the Foodborne Diseases Active Surveillance Network (FoodNet) and the Tennessee Department of Health (TDH). These data included demographic and exposure data for Tennessee residents who were diagnosed with a culture-confirmed *Salmonella* infection and interviewed from January 2013 through December 2015.

### 2.2. Study Design

A case-case analysis was conducted. Case-case comparisons use existing case data as the “control” group. “Cases” were defined as a person diagnosed with a culture-confirmed NTS infection caused by any of the three *Salmonella* serotypes of interest, *S.* Newport, *S*. Javiana, and *S.* Mississippi, from January 2013 through December 2015 in Tennessee. The comparison group, referred to as the “control” group, was defined as a person diagnosed with culture-confirmed salmonellosis with serotypes other than *S.* Newport, *S.* Javiana, and *S.* Mississippi during the same time frame in Tennessee.

Exposure data were collected by interviews performed using a standard *Salmonella* questionnaire. In total, 254 exposure variables were included in the standard questionnaire. All patients were asked about food and water sources, person-to-person contact, and exposure to animals in the seven days prior to the onset of illness. Standard food histories included questions about restaurants and consumption of fruits, vegetables, meats, fish and seafood, frozen ready-to-eat foods, and dairy, as well as poultry products. Questions about sources of drinking water and recreational water exposures were routinely asked. Cases were asked about attendance at festivals, concerts, sporting events, reunions, and/or religious gatherings seven days prior to the onset of disease. Animal exposure questions included direct or indirect contact with live animals; and contact with pets, pet foods, manure, and compost.

### 2.3. Data Cleaning

TDH data were reviewed and cleaned by running range checks on dates, frequencies, text, categorical variables, and continuous variables using SAS Version 9.4 (SAS Institute Inc., Cary, NC, USA).

### 2.4. Statistical Analysis

To assess potential selection bias, the categorical demographic variables among case and control groups were compared descriptively and with chi-square tests. The continuous variable, age, was categorized following the standard categorization of *Salmonella* infection used by FoodNet [20]. Logistic regression (PROC LOGISTIC) was performed to determine the association between having one of the three selected serotype infections and various exposures. The crude odds ratio (OR), 95% confidence intervals (CI), and *P*-values are presented.

To adjust for potential confounding, multivariable logistic regression analyses were performed. The regions were classified as three greater divisions of East, Middle, and West Tennessee. Some exposure variables were created by combining multiple items from the questionnaire. For example, the consumption of “any tomatoes” was constructed by combining “cherry tomatoes,” “grape tomatoes,” “Roma tomatoes,” “other (e.g., beefsteak) tomatoes”, and “sold on vine tomatoes.” Similarly, the consumption of “any cheese” was constructed by combining all the cheese types mentioned in the questionnaire. Likewise, “contact with any animals” was constructed by combining all types of animal exposure information available in the questionnaire. Due to the very large number of exposures in the dataset, only those variables with *P* ≤ 0.2 were selected for potential inclusion in the model. To identify potential confounders, each demographic variable, such as gender, race, ethnicity, age, and region, was entered into the model with the exposure variable, one at a time. If the variable changed the OR by >10%, it remained in the model. Additional variables were added until the estimate no longer changed by >10%. The adjusted OR controlling for demographic variables, 95% CI, and *P*-value are presented. To identify those exposures associated with the three selected serotypes, even when adjusted for all other exposures, we created one final multivariable that included all risk factors with *P* ≤ 0.1 and all demographic variables. All data analyses were performed using SAS version 9.4 (SAS Institute Inc., Cary, NC, USA).

## 3. Results

**Figure 1:**
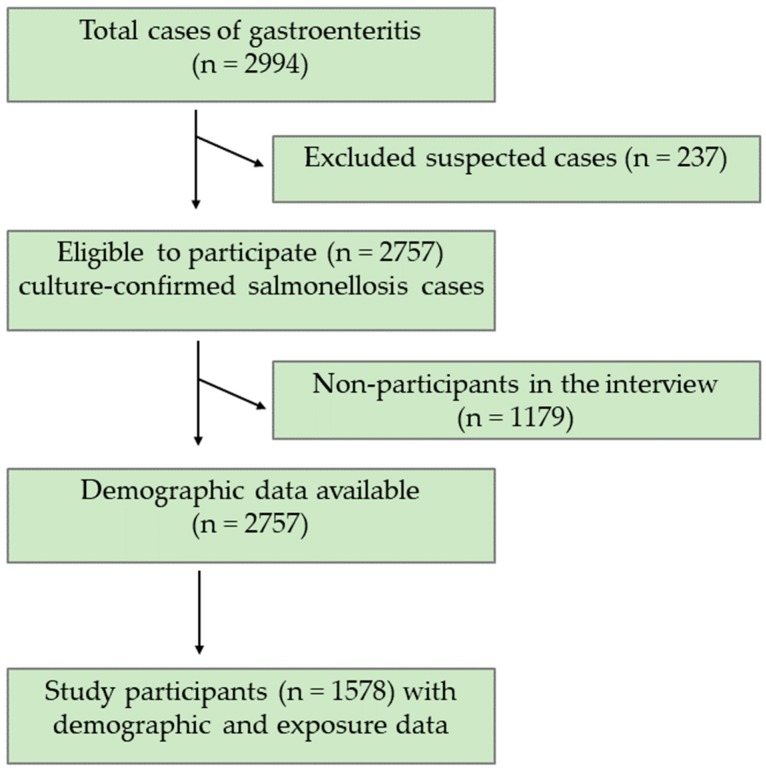
Consort Diagram of study participants who completed the interview.

A total of 2757 (92%) culture-confirmed salmonellosis cases were included and 237 (8%) suspected salmonellosis cases were excluded from the analysis (Figure 1). Demographic data were available for all 2757 culture-confirmed salmonellosis cases. The *Salmonella*-infected patients were in the age range of <1 year to 97 years. Exposure data were available for 1578 of 2757 culture-confirmed salmonellosis patients. Of the 2757 *Salmonella*-infected patients, 640 (23%) patients were infected with *S.* Newport, *S.* Javiana, or *S.* Mississippi, and, thus, were considered as a “case”. More cases were female (*n* = 333, 52%), white (*n* = 483, 84%), or non-Hispanic (*n* = 570, 98%) and the most common age group was adults aged 61 years and above (*n* = 135, 21%) (Table 1). A majority of the cases (*n* = 342, 53%) were reported from the West Grand Region of Tennessee. Among the cases, the most prevalent *Salmonella* serotype was *S*. Newport (*n* = 299, 47%), followed by *S*. Javiana (*n* = 239, 37%), and *S*. Mississippi (*n* = 102, 16%) (Table 1).

Of the 2757 lab-confirmed *Salmonella*-infected patients, 1578 (57%) patients had a completed interview that was performed using the standard *Salmonella* questionnaire (Table 2). The odds for participation were 60% higher in two age groups, (a) below 1 year and (b) 41–60 years, as compared to the participants aged 21–40 years. Likewise, the odds for participation was 50% higher in the age group, 61 years and above. The residents of West Grand Region, as well as the cases, were significantly less likely to participate in the interview (Table 2).

The demographics of the study participants are described in Table 3. Of the 1578 study participants, 331 (21%) were infected with one of the three *Salmonella* serotypes, namely, *S.* Newport, *S.* Javiana, and *S.* Mississippi. The majority (*n* = 157, 47%) of the cases were identified from the West Grand Region. *S*. Newport was identified as the predominant (*n* = 170, 51%) *Salmonella* serotype among the three selected case-associated serotypes (Table 3).

To identify the exposures associated with the three selected *Salmonella* serotypes, exposure status was compared between the case and comparison groups. Table 4 presents the crude and adjusted ORs, as well as 95% CI for those exposures with *P*-value ≤ 0.2. There was a significantly increased risk, adjusted for age, among those who consumed frozen pizza seven days before the onset of illness (OR _age-adjusted_ = 1.4 (95% CI 1.0, 1.9), *P* = 0.02) and those who consumed powdered-formula baby food (OR _age and gender-adjusted_ = 1.7 (95% CI 1.1, 2.4), *P* < 0.01) (Table 4). This study showed an increased risk for cases who had contact with dogs (OR = 1.3 (95% CI 1.0, 1.7), *P* = 0.02). In addition, this study found a significantly elevated odds ratio controlling for the region of residence for the exposure “visit to a farm a week prior to the onset of disease” (OR _region-adjusted_ = 2.2 (95% CI 1.2, 3.7), *P* < 0.01). Exposure with pet treats or chews seven days before illness was associated with a significantly increased risk while controlling for age, race, and region of residence (OR = 1.7 (95% CI 1.2, 2.3), *P* < 0.01) (Table 4).

The final multivariable model identified that exposure to pet treats/chews (adjusted OR = 3.0 (95% CI 1.6, 5.5), *P* = 0.0005) was significantly associated with the cases (Supplementary Table S1). In this analysis, all other exposures were not found to be significantly associated (data presented in Supplementary Table S1). The consumption of powdered baby formula food and store-bought pureed baby food were risk factors in the previous model (Table 4); however, in the multivariable model (Supplementary Table S1), these exposures were not significantly associated with the cases after adjusting for all demographic and exposure variables.

## 4. Discussion

In this study, we extensively analyzed the exposure information of more than 250 exposure variables to identify potential exposures associated with *S.* Newport, *S*. Javiana, and *S*. Mississippi in NTS cases in Tennessee. Among all these variables, we found exposures to pet treats or chews as a significant risk factor for all of the case-associated NTS infections. Previous studies [21,22,23,24,25,26] have demonstrated dogs and pet treats as potential risk factors for human NTS infections. Pet foods were reported to be associated with an *S*. Newport outbreak in humans [26]. In addition to *S*. Newport, many other *Salmonella* serotypes, including *S.* Infantis, *S.* Typhimurium, and *S.* Derby, were isolated from contaminated pet treats [22,23,24,26]. Although there are several types of pet treats available commercially, most of them are made from animal body parts, such as pig ears and cow hooves; hence, contamination may occur if the pet treat is prepared from a contaminated animal origin.

There were other exposures that were found to have more than 10% OR changes, even though they did not result in statistical significance in our analysis. For example, the current study revealed that the consumption of frozen pizza and powdered baby formula food as risk factors for NTS infections caused by any of the case-associated serotypes. Frozen pizza has been previously reported to be associated with several foodborne pathogens, including *Escherichia coli*, *Listeria*, and *Salmonella* [27]. The present study identified that the consumption of powdered formula was associated with the cases. Although NTS infection in infants is observed frequently, [28] little is known about risk factors in this high-risk population. Risk factors associated with NTS infections in infants or children are more likely to be different from the other age group population since these risk factors are based on their food sources, eating habits, and the stage of their immune system development [29].

The present study also identified some exposures that could be conceived as the so-called “protective factors” (used commonly in epidemiological studies) for *S*. Newport, *S*. Javiana, and *S*. Mississippi. In this case-case format, such exposures may be important transmission factors for the comparison group. We observed that most of the “protective” exposures for the case group were foodborne exposures. This finding is consistent with previous literature, indicating that many of the serotypes in the comparison group were largely foodborne *Salmonella* serotypes that were implicated in foodborne outbreak or illnesses [2,3,30]. In this study, the consumption of several types of fruits and vegetables was associated with the comparison group. The consumption of tomato and lettuce was described in the literature as risk factors for *S.* Newport [31,32] and *S*. Javiana [33,34,35] infections. In contrast to the previously reported studies, current findings demonstrated that the consumption of raw tomato and lettuce was less likely to be associated with *S.* Newport, *S*. Javiana, and *S.* Mississippi infections. Several studies identified the consumption of cheese [36,37] and eating out at a seafood restaurant [38] as risk factors for *S*. Javiana infection, whereas the present study contradicts these findings. The contradictory findings in our study do not necessarily mean that tomato, lettuce, and cheese are not potential carriers for *S.* Newport, *S*. Javiana, and *S.* Mississippi infections, given that any of the mentioned food items may carry diverse *Salmonella* serotypes.

The present study demonstrated that the cases were more likely to have been exposed to animals than the comparison group. This finding is expected since direct or indirect contact with an animal has previously been identified as a risk factor for NTS infections [7,8,9]. The current study identified that visiting a farm a week before the illness was associated with the cases. Several studies support the current study findings since cattle are considered as primary carriers of *S*. Newport [39,40]. Thus, direct contact with cattle or farm animals, as well as occupational exposure, is considered to be a threat to the transmission of *S*. Newport infection in humans [20,41]. Thus, touching and handling pet foods pose a risk for human NTS infections. Therefore, appropriate hand hygiene is needed to eliminate the chances of cross-contamination while handling pet foods. In addition, the ingestion of improperly cooked beef contaminated during the slaughtering process can cause foodborne salmonellosis [42] and contact with animal feces may also result in the contamination of fertilizers and, thus, the bacteria can spread to fresh produce [43].

The results of this study should be interpreted, keeping in mind the following limitations. Fundamentally, the comparison group is not a well-controlled group in population-based case-control studies. Both the case and the comparison groups were diagnosed with salmonellosis, but they differed by serotype. The controls were *Salmonella*-infected patients and they likely did not represent the exposure prevalence in the general population. Additionally, there were significant differences observed in the completion of interviews by region of the state. Lastly, the routine questionnaire used for *Salmonella* cases was lengthy, and the person answering might have lost interest in answering all the questions. This may lead to a non-differential misclassification bias since the exposure is equally misclassified in both cases and comparison groups. Similarly, both cases and comparison group may equally misclassify the foodborne exposure status in the interview, because, the gap between the onset of disease and the interview may sometimes be more than a week. Therefore, it is sometimes hard to remember what food they have had a week before disease onset, which is approximately two weeks before the interview, unless the same person is either sensitive to a particular food or developed a strong aversion against a food item. Therefore, this may cause information bias, more specifically, a non-differential misclassification in exposure status.

## 5. Conclusions

The results of the present study suggested that human NTS infections caused by any of the three serotypes, namely, *S.* Newport, *S*. Javiana, and *S*. Mississippi were significantly associated with pet treats or chews in adjusted univariate models. We also found that animal exposures, such as visit to a farm and contact with a dog, had a higher odds ratio of contracting NTS infections. In addition to animal exposures, this study pointed out that very few foodborne exposures were associated with the cases. This study agrees with previous studies which demonstrated that *S.* Newport, *S*. Javiana, and *S*. Mississippi infections are mostly associated with animal or environmental exposures. Educating the public about the modes of transmission of the disease could reduce risks.

## Figures and Tables

**Table 1:** Characteristics of the Tennessee salmonellosis cases.

Variables		Case, n * = 640 (%)	Comparison Group, n * = 2117 (%)	OR (95% CI)	*P*-Value
Gender	Female	333 (52)	1106 (53)	Reference	Reference
	Male	306 (48)	996 (47)	1.0 (0.8, 1.2)	0.82
Race	White	483 (84)	1525 (84)	1.0 (0.8, 1.3)	0.70
	Others	91 (16)	298 (16)	Reference	Reference
Ethnicity	Hispanic	9 (2)	66 (4)	Reference	Reference
	Non-Hispanic	570 (98)	1739 (96)	2.4 (1.2, 4.8)	0.01
Age (years)	<1	86 (13)	168 (8)	2.3 (1.6, 3.2)	<0.0001
	1–4	112 (18)	304 (14)	1.6 (1.2, 2.2)	<0.01
	5–12	68 (11)	212 (10)	1.4 (1.0, 2.1)	0.05
	13–20	38 (6)	147 (7)	1.2 (0.8, 1.8)	0.50
	21–40	83 (13)	369 (18)	Reference	Reference
	41–60	118 (18)	448 (21)	1.2 (0.9, 1.6)	0.30
	≥61	135 (21)	464 (22)	1.3 (1.0, 1.8)	0.09
Region	East Grand Region	151 (24)	736 (35)	Reference	Reference
	Middle Grand Region	147 (23)	811 (38)	0.9 (0.7, 1.1)	0.32
	West Grand Region	342 (53)	565 (27)	3.0 (2.4, 3.7)	<0.0001
Year	2013	174 (27)	698 (33)	Reference	Reference
	2014	266 (42)	697 (33)	1.5 (1.2, 1.9)	<0.001
	2015	200 (31)	717 (34)	1.1 (0.9, 1.4)	0.30
Serotype	*S*. Newport	299 (47)	0 (0)	-	-
	*S*. Javiana	239 (37)	0 (0)	-	-
	*S*. Mississippi	102 (16)	0 (0)	-	-

Case = *Salmonella* Newport, *Salmonella* Javiana, *Salmonella* Mississippi; comparison group = other *Salmonella* serotypes, except for the three selected case-associated serotypes; others = African American, Asian, American Indian, Alaska Native, Native Hawaiian, or Other Pacific Islander; reference = variable used to calculate odds ratio. * Data for some demographic variables were not always available; only available data are presented and used for percentage calculations.

**Table 2:** Comparing participants and non-participants that responded to the *Salmonella* questionnaire.

Variables		Participants, n * = 1578 (%)	Non-Participants, n * = 1179 (%)	OR (95% CI)	*P*-Value
Gender	Female	874 (56)	566 (48)	Reference	Reference
	Male	696 (44)	610 (52)	0.7 (0.6, 0.9)	<0.0001
Race	White	1285 (88)	725 (77)	2.3 (1.8, 2.8)	<0.0001
	Others	171 (12)	220 (23)	Reference	Reference
Ethnicity	Hispanic	44 (3)	32 (3)	Reference	Reference
	Non-Hispanic	1376 (97)	937 (97)	1.1 (0.7, 1.7)	0.70
Age (years)	<1	158 (10)	96 (8)	1.6 (1.2, 2.2)	<0.001
	1–4	230 (15)	188 (16)	1.2 (0.9, 1.5)	0.24
	5–12	155 (10)	125 (11)	1.2 (0.9, 1.6)	0.20
	13–20	88 (5)	97 (8)	0.9 (0.6, 1.2)	0.41
	21–40	231 (15)	221 (19)	Reference	Reference
	41–60	352 (22)	215 (18)	1.6 (1.2, 2.0)	<0.0001
	≥61	364 (23)	237 (20)	1.5 (1.2, 1.9)	<0.001
Region	East Grand Region	545 (35)	344 (29)	Reference	Reference
	Middle Grand Region	621 (39)	337 (29)	1.2 (1.0, 1.4)	0.11
	West Grand Region	412 (26)	498 (42)	0.5 (0.4, 0.6)	<0.0001
Year	2013	446 (28)	426 (36)	Reference	Reference
	2014	617 (39)	349 (30)	1.7 (1.4, 2.0)	<0.0001
	2015	515 (33)	404 (34)	1.2 (1.0, 1.5)	0.03
Case	Comparison group	1246 (79)	866 (74)	Reference	Reference
	Cases	331 (21)	309 (26)	0.8 (0.6, 0.9)	0.001

Case = *S*. Newport, *S*. Javiana, *S*. Mississippi; comparison group = other *Salmonella* serotypes, except for the three selected case-associated serotypes; others = African American, Asian, American Indian, Alaska Native, Native Hawaiian, or Other Pacific Islander; reference = variable used to calculate odds ratio. * Data for some demographic variables were not always available; only available data are presented and used for percentage calculations.

**Table 3:** Demographics of the participants in the case-case study.

Variables		Case, n * = 331 (%)	Comparison Group, n * = 1246 (%)	OR (95% CI)	*P*-Value
Gender	Female	171 (52)	703 (57)	Reference	Reference
	Male	159 (48)	536 (43)	1.2 (1.0, 1.5)	0.11
Race	White	276 (88)	1008 (88)	0.9 (0.6, 1.4)	0.70
	Others	39 (12)	132 (12)	Reference	Reference
Ethnicity	Hispanic	5 (2)	39 (4)	Reference	Reference
	Non-Hispanic	304 (98)	1071 (96)	2.2 (0.9, 5.7)	0.10
Age (years)	<1	49 (15)	109 (9)	2.5 (1.5, 4.1)	<0.001
	1–4	58 (18)	171 (14)	1.9 (1.2, 3.0)	<0.01
	5–12	40 (12)	115 (9)	2.0 (1.2, 3.2)	0.01
	13–20	11 (3)	77 (6)	0.8 (0.4, 1.6)	0.50
	21–40	35 (10)	196 (16)	Reference	Reference
	41–60	66 (20)	286 (23)	1.3 (0.8, 2.0)	0.20
	≥61	72 (22)	292 (23)	1.4 (0.9, 2.1)	0.10
Region	East Grand Region	82 (25)	462 (37)	Reference	Reference
	Middle Grand Region	92 (28)	529 (42)	1.0 (0.7, 1.4)	0.90
	West Grand Region	157 (47)	255 (21)	3.5 (2.6, 4.7)	<0.0001
Year	2013	80 (24)	366 (29)	Reference	Reference
	2014	154 (47)	462 (37)	1.5 (1.1, 2.0)	<0.001
	2015	97 (29)	418 (34)	1.1 (0.8, 1.5)	0.72
Serotype	*S*. Newport	170 (51)	0 (0)	-	-
	*S*. Javiana	116 (35)	0 (0)	-	-
	*S*. Mississippi	45 (14)	0 (0)	-	-

Case = *S*. Newport, *S*. Javiana, *S*. Mississippi; comparison group = other *Salmonella* serotypes, except for the three selected case-associated serotypes; others = African American, Asian, American Indian, Alaska Native, Native Hawaiian, or Other Pacific Islander; reference = variable used to calculate odds ratio. * Data for some demographic variables were not always available; only available data are presented and used for percentage calculations.

**Table 4:** Selected exposures associated with the three *Salmonella* serotypes, *S.* Newport, *S*. Javiana, and *S.* Mississippi, from Tennessee, USA, from 2013 through 2015.

Exposure	Unadjusted OR (95% CI)	Adjusted OR (95% CI)	*P*-Value ^@^
Consumed dairy and poultry products in the 7 days prior to illness			
Any cheese	0.7 (0.5, 0.9)		<0.01
Processed sliced cheese	0.8 (0.6, 1.1)		0.11
String cheese	0.7 (0.4, 1.1)		0.16
Cottage cheese	0.4 (0.2, 0.8)		0.01
Fresh/dried Parmesan/Romano/or similar cheese	0.5 (0.3, 0.8)		0.01
Eggs	0.6 (0.4, 0.9)		0.03
Ice cream	1.3 (0.9, 1.7)		0.09
Whole chicken	0.7 (0.6, 0.9)		0.02
Consumed frozen foods in the 7 days prior to illness			
Frozen pizza		1.4 * (1.0, 1.9)	0.02
Ate out at restaurants in the 7 days prior to illness			
Ate out at Mexican/Tex-Mex restaurants	0.7 (0.4,1.0)		0.10
Ate out at Seafood restaurants	0.5 (0.2, 1.0)		0.05
Consumed fish and seafood in the 7 days prior to illness			
Ate any type of fish or fish products	0.7 (0.5, 0.9)		0.02
Consumed vegetables in the 7 days prior to illness			
Asparagus	0.5 (0.3, 0.9)		0.03
Avocados	0.7 (0.4, 1.2)		0.20
Broccoli	0.7 (0.5, 1.0)		0.08
Bell peppers (green/red/yellow/orange)	0.6 (0.4, 0.9)		<0.01
Carrots	0.8 (0.5,1.0)		0.11
Fresh herbs or spices (e.g., basil, parsley, and cilantro)	0.5 (0.3, 1.0)		0.04
Fresh lemon or lime (including any garnishes in drinks)	0.7 (0.4, 1.0)		0.04
Hot chili/chili peppers (e.g., jalapeños or seranos)	0.5 (0.2, 0.9)		0.04
Lettuce or other greens (including on a sandwich)	0.7 (0.5, 1.0)		<0.01
Green onions	0.6 (0.3, 1.1)		0.11
White or yellow onions	0.7 (0.5, 0.9)		0.01
Potatoes	0.7 (0.504, 0.9)		<0.01
Salsa or pico de gallo	0.7 (0.4, 1.0)		0.06
Any tomatoes	0.8 (0.6, 1.0)		0.04
Grape tomatoes	0.4 (0.2, 1.2)		0.11
Roma tomatoes	0.6 (0.3, 1.2)		0.15
Tomatoes sold on vine	0.3 (0.1, 0.5)		<0.0001
Consumed fruits in the 7 days prior to illness			
Apples	0.8 (0.6, 1.0)		0.12
Banana	0.8 (0.6, 1.0)		0.12
Blackberries	0.6 (0.3, 1.1)		0.09
Grapefruit	0.5 (0.2, 1.4)		0.2
Mango	0.3 (0.1, 0.9)		0.02
Pineapple	0.6 (0.4, 1.0)		0.03
Tangerines	0.5 (0.2, 0.9)		0.03
Consumed nuts and seeds in the 7 days prior to illness			
Almonds	0.7 (0.4, 1.1)		0.11
Cashews	0.5 (0.3, 0.9)		0.03
Whole peanuts	0.7 (0.5, 1.1)		0.13
Consumed baby foods in the 7 days prior to illness			
Powdered baby formula		1.7 ** (1.1, 2.4)	<0.01
Store-bought pureed baby food (e.g., Gerber)		1.5 ** (0.9, 2.4)	0.10
Water exposure in the 7 days prior to illness			
Source of water at school/work—do not use tap water	0.4 (0.2, 0.9)		0.02
Source of water at school/work—well water		2.9 ^†^ (0.8, 10.7)	0.11
Recreational water exposure	1.3 (0.9, 1.7)		0.18
Contact with a live animal, pet, and pet food in the 7 days prior to illness			
Visit to a farm		2.2 ^#^ (1.2, 3.7)	<0.01
Contact with any animal	1.2 (0.9, 1.4)		0.15
Contact with a mammal	1.2 (0.9, 1.5)		0.17
Contact with a dog	1.3 (1.0, 1.7)		0.02
Contact with a tropical fish or aquariums	0.5 (0.2, 1.0)		0.07
Contact with pet treats or chews		1.7 *** (1.2, 2.3)	<0.01

* Age-adjusted OR; ** age- and gender-adjusted OR; *** age-, race-, and region-adjusted OR; ^†^ age-, race-, region-, and ethnicity-adjusted OR; ^#^ region-adjusted OR; ^@^ exposures with *P* < 0.2 are shown here.

## Supplementary Materials

The following are available online at https://www.mdpi.com/2076-0817/9/2/78/s1, Table S1: Multivariable analysis presenting adjusted OR controlling for all demographic and exposure variables.
