# Supplementary material for: Exposures Associated with Non-Typhoidal Salmonella Infections Caused by Newport, Javiana, and Mississippi Serotypes in Tennessee, 2013–2015: A Case-Case Analysis
Source: Pathogens. 2020 Jan 24;9(2):78. doi: 10.3390/pathogens9020078 (PMC7168582; doi:10.3390/pathogens9020078)
Supplement: Supplementary file 1 [file pathogens-09-00078-s001.pdf]

Table S1. Multivariable analysis presenting adjusted OR controlling for all demographic and exposure variables.

Exposure	Adjusted OR (95% CI)	*P* value
Consumed dairy and poultry products in the 7 days prior to illness		
Any cheese	0.8 (0.5, 1.2)	0.31
Cottage cheese	0.9 (0.3, 2.5)	0.88
Fresh/dried Parmesan/Romano/or similar cheese	0.5 (0.2, 1.3)	0.14
Eggs	0.8 (0.4, 1.6)	0.52
Ice cream	1.5 (0.9, 2.4)	0.10
Whole chicken	0.8 (0.5, 1.3)	0.38
Consumed frozen foods in the 7 days prior to illness		
frozen pizza	1.5 (0.9, 2.5)	0.11
Ate out restaurants in the 7days prior to illness		
Ate out at Mexican/Tex–Mex restaurants	0.8 (0.4, 1.9)	0.69
Ate out at Seafood restaurants	0.3 (0.1, 1.1)	0.07
Consumed fish and seafood in the 7 days prior to illness		
Ate any type of fish or fish products	0.8 (0.3, 2.1)	0.69
Consumed vegetables in the 7 days prior to illness		
Asparagus	0.7 (0.2, 2.1)	0.54
Broccoli	1.2 (0.6,2.2)	0.61
Bell peppers (green/red/yellow/orange)	0.8(0.4, 1.6)	0.56
Fresh herbs or spices (e.g., basil, parsley, cilantro)	0.4 (0.06, 2.6)	0.33
Fresh lemon or lime (including any garnishes in drinks)	1.4 (0.6, 3.2)	0.45
Hot chili/chile peppers (e.g., jalapeños or seranos)	0.5 (0.1, 2.0)	0.31
Lettuce or other greens, including on a sandwich	1.1 (0.6, 1.8)	0.80
White or yellow onions	0.8 (0.5, 1.5)	0.51
Potatoes	0.7 (0.4, 1.2)	0.16
Salsa or pico de gallo	0.9 (0.4,1.9)	0.73
Any raw tomatoes	1.1(0.7, 1.9)	0.63
Raw tomato sold on vine	0.3 (0.1,1.0)	0.05
Consumed fruits in the 7 days prior to illness		
Blackberries	0.5 (0.1, 1.8)	0.29
Pineapple	0.6 (0.2, 1.4)	0.22
Tangerines	0.4 (0.1, 1.2)	0.10
Consumed nuts and seeds in the 7 days prior to illness		
Cashews	0.4 (0.2, 1.1)	0.09
Consumed baby foods in the 7 days prior to illness		
Powdered baby formula	1.3 (0.5, 3.2)	0.62
Store-bought pureed baby food (e.g., Gerber)	0.8 (0.3, 1.8)	0.56
Water exposure in the 7days prior to illness		
Source of water at School/work—Do not use Tap water	0.2 (0.1, 0.7)	0.01
Source of water at School/work—Private well water	0.8 (0.09, 6.4)	0.80
Recreational water exposure	1.7 (0.9, 3.0)	0.08

**Exposure**	**Adjusted OR (95% CI)**	***P* value**
Contact with live animal, pet and pet food in the 7days prior to illness		
Visit to a farm	2.3 (0.9, 6.0)	0.08
Contact with any animals	0.7 (0.4,1.3)	0.28
Contact with mammal	1.2 (0.7, 2.3)	0.49
Contact with dog	1.6 (0.9,2.7)	0.09
Contact with tropical fish or aquariums	0.4 (0.1, 1.3)	0.13
Contact with pet treats or chews	3.0 (1.6,5.5)	0.0005
